# Infant Appendicitis: A Case Presentation of Appendicitis in a Nine-Month-Old Infant With Respiratory Syncytial Virus (RSV) and Otitis Media and Review of Literature

**DOI:** 10.7759/cureus.56059

**Published:** 2024-03-12

**Authors:** Leslie A Hueschen, April McNeill-Johnson

**Affiliations:** 1 Pediatric Emergency Medicine, Children's Mercy Hospital/University of Missouri-Kansas City, Kansas City, USA

**Keywords:** case report, complications, pediatric, infant, appendicitis

## Abstract

Appendicitis in children < 5 years old is uncommon and even less common in children < 1 year old. Symptoms of appendicitis can be non-specific and mimic other common pediatric diseases, causing delays in diagnosis. Without a timely diagnosis, young children with appendicitis are at risk of developing worsening disease, intra-abdominal abscess, perforation, and bacteremia.

We submit a case of a nine-month-old male infant presenting with fever, vomiting, and irritability seen the day prior and treated for otitis media, who was ultimately diagnosed with appendicitis with concomitant viral infection (respiratory syncytial virus and human rhinovirus/enterovirus) and treated with intravenous antibiotics and laparoscopic appendectomy.

This case illustrates how easy it is to misdiagnose infant appendicitis due to factors including normal developmental speech barriers, nonspecific presentations, and overlap of symptoms with many other common childhood illnesses, along with a challenging abdominal examination. Delay in diagnosis leads to increased rates of perforation and complications. Providers should trust abnormal physical examination findings, especially abdominal guarding against palpation, and keep a wide differential diagnosis in order to diagnose appendicitis in young children.

## Introduction

Abdominal pain is a common pediatric presenting complaint in the emergency department (ED). Symptoms of appendicitis are non-specific, but the history of periumbilical and right lower quadrant pain, paired with physical examination findings of abdominal guarding and signs of peritonitis, usually helps guide providers to the final diagnosis of appendicitis. Appendicitis incidence peaks in the second decade of life. Appendicitis in children < 5 years old is uncommon (<9%) and even less common in children < 1 year old (<0.9%) [1]. It is rarely included in the differential diagnosis of young children presenting with fever and vomiting and is commonly misdiagnosed [2]. Here, we present a case of a nine-month-old presenting with fever and vomiting with a diagnosis of otitis media and viral upper respiratory tract infection (URI), from respiratory syncytial virus (RSV) and human rhinovirus/enterovirus, who was ultimately diagnosed with appendicitis and treated with intravenous (IV) antibiotics and appendectomy.

## Case presentation

This is a case of a nine-month-old immunized, previously healthy male infant who presented with complaints of vomiting. The patient's diet consisted of formula and developmentally appropriate solid foods. He was reported as having normal stooling habits and prior to his illness had no history of constipation. The patient started feeling unwell two days prior. He had a low-grade fever and was not treated with antipyretics at home. The patient developed non-bilious, non-bloody vomiting 24 hours before arriving at the ED, progressing to vomiting after every feed and dry heaving between feeds. The day prior to presentation, the patient was seen at their primary care pediatrician's office, where they were diagnosed with otitis media and given 1000 mg of ceftriaxone intramuscularly. The patient's mother tried feeding the patient electrolyte solution and water overnight, but the patient continued to vomit with decreased urine output. The review of systems was negative for cough, congestion, runny nose, difficulty breathing, constipation, and diarrhea.

On arrival, the patient's vitals showed a temperature of 39.6°C, heart rate of 208, respiratory rate of 48, SpO2 of 96%, and pain score of 7/10 (based on infant FLACC pain score). The physical examination was notable for tachycardia with a capillary refill of two to three seconds. The patient was alert but fussy. The lungs were clear to auscultation, without crackles or wheezing. The abdomen was nondistended and tender to palpation especially in the right lower quadrant with rebound tenderness and guarding. He had a normal genital examination for his age. Ear examination demonstrated erythema, without bulging, of the left tympanic membrane and an inability to visualize the right tympanic membrane.

With the combination of guarding on abdominal examination, fever, tachycardia, and vomiting, the working differential included sepsis, viral gastroenteritis, viral syndromes, urinary tract infection, viral URI, dehydration, and appendicitis. Initial work-up included abdominal ultrasound (US), respiratory viral testing, blood culture, complete blood count with differential, C-reactive protein (CRP), basic metabolic panel (BMP), urine analysis, and urine culture. The patient was given an IV of 20 ml/kg normal saline fluid bolus and ceftriaxone. The ultrasound did not clearly visualize the appendix and did not show any abnormal echogenic mesenteric fat or mass. There was no free fluid or abscess, and no abnormal lymph nodes were identified. All structures in the right lower quadrant demonstrated compressibility. The laboratory results are described below (Table 1).

**Table 1:** Patient laboratory results

Laboratory Values	Patient Results	Normal Reference Range
WBC (x10^3/mcL)	17.92 x 10 3	6-17.5
Hemoglobin (gm/dL)	13 gm/dL	10.5-13.5
Hematocrit (%)	38.5	33-39
Platelet (x10^3/mcL)	230	150-450
Absolute Immature Granulocytes (x10^3/mcL)	0.05	0.0-0.04
Absolute neutrophil count (x10^3/mcL)	12.24	1.5-8.5
Absolute lymphocyte count (x10^3/mcL)	3.49	4-10
Absolute monocyte count (x10^3/mcL)	1.83	0.02-1.8
Absolute eosinophil count (x10^3/mcL)	0.23	0.0-.06
Absolute basophil count (x10^3/mcL)	0.08	0.0-0.1
Sodium (mmol/L)	142	135-145
Potassium (mmol/L)	4.6	3.5-5.2
Chloride (mmol/L)	108	99-112
Carbon dioxide (mmol/L)	16	20-30
Anion gap (mmol/L)	18	7-14
Calcium (mg/dl)	9.9	8.6-10.5
Glucose (mg/dL)	92	60-110
BUN (mg/dL)	10	5-20
Creatinine (mg/dL)	0.26	0.06-0.45
C-reactive protein (mg/dL)	2.8	0.0-1.0
Urine specific gravity	>1.050	1.005-1.035
Urine pH	5.0	4.6-8
Urine glucose	Negative	Negative
Urine protein	Negative	Negative
Urine ketones	2+	Negative
Urine blood	1+	Negative
Urine bilirubin	Negative	Negative
Urine nitrite	Negative	Negative
Urine leukocyte esterase	Negative	Negative
Urine WBC (/hpf)	1-4	1-4
Urine RBC (/hpf)	5-15	1-4
Urine bacteria (/hpf)	None	None

**Figure 1:**
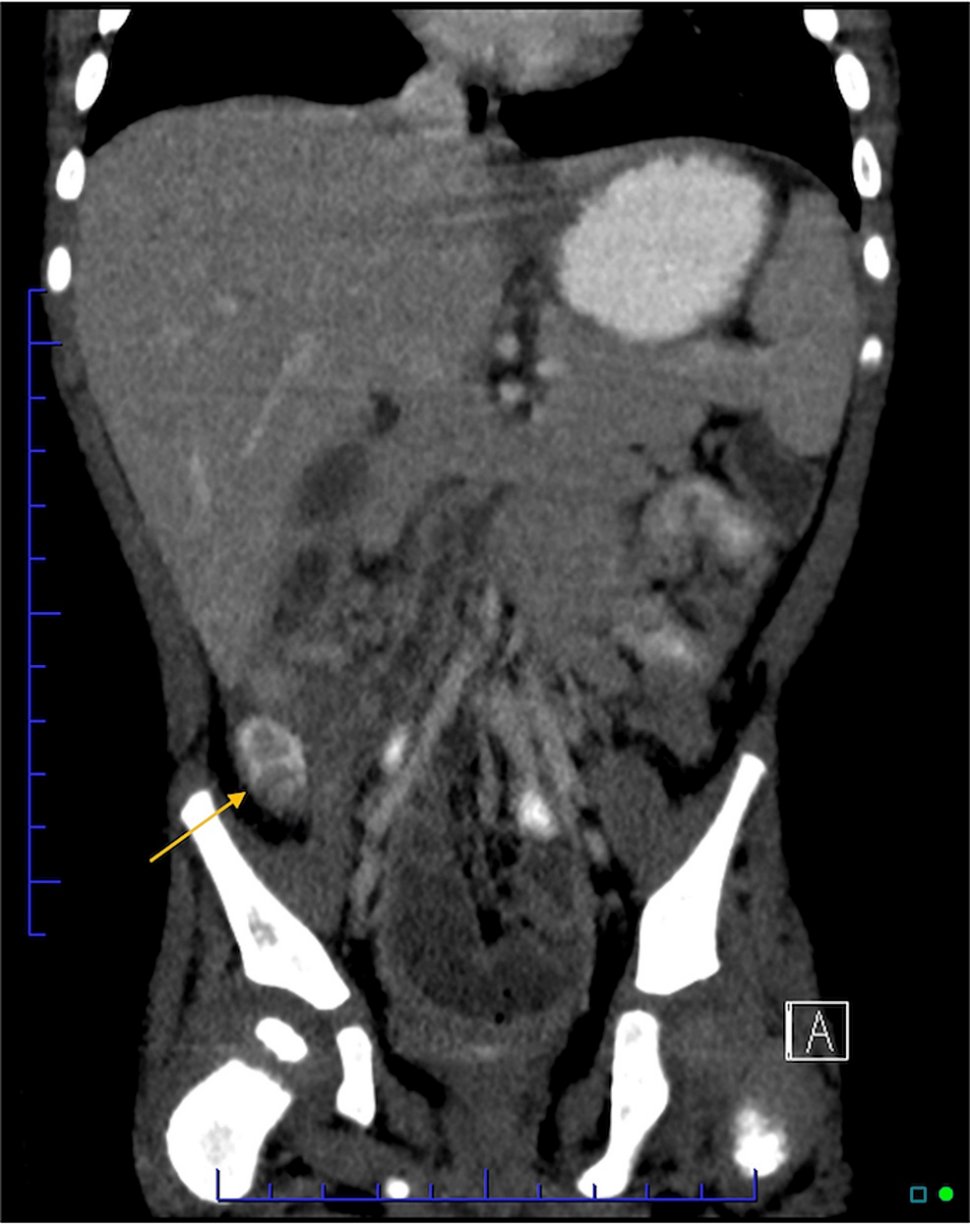
CT Abdomen/pelvis with IV and oral contrast: Demonstrating a fluid-filled tubular structure, prominent in size, in the right hemiabdomen likely to be retro-ileal appendix with adjacent inflammation/fluid, favored to represent appendicitis (coronal section)

**Figure 2:**
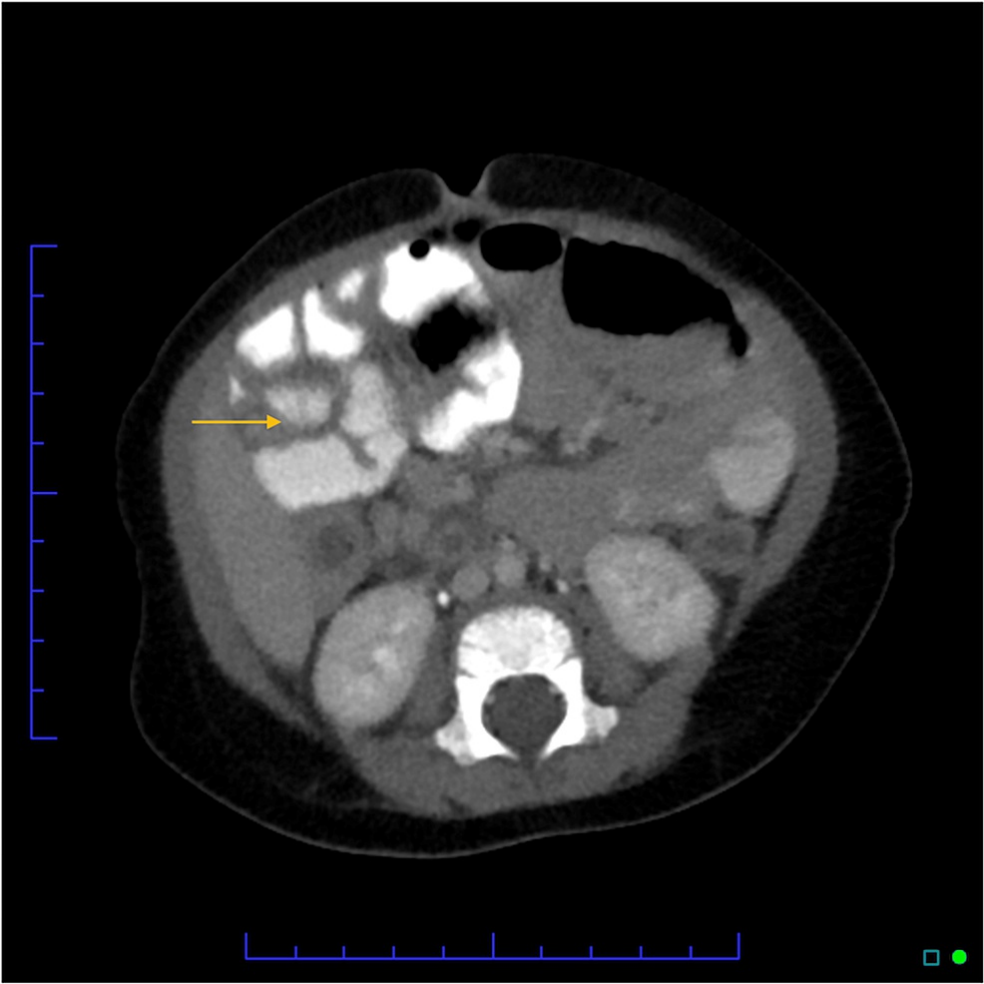
CT Abdomen/pelvis with IV and oral contrast: Demonstrating a fluid-filled tubular structure, prominent in size, in the right hemiabdomen likely to be retro-ileal appendix with adjacent inflammation/fluid, favored to represent appendicitis (transverse section)

The patient was given acetaminophen upon arrival due to fever and fussiness. Serial abdominal examinations over four hours while the patient was sleeping demonstrated continued right lower quadrant abdominal pain with palpation, causing the patient to wake up and cry. In the interim, respiratory viral testing was positive for human rhinovirus/enterovirus and RSV. Despite acetaminophen, IV fluids, and tolerating oral fluids, the patient continued to be ill-appearing on the exam and would cry with any abdominal palpation. The continued abdominal pain and ill appearance, combined with a lack of upper respiratory tract symptoms, made us concerned for possible appendicitis, providers pursued an abdominal computed tomography (CT) with IV and oral contrast. CT showed a fluid-filled tubular structure, prominent in size, in the right hemiabdomen likely to be retro-ileal appendix with adjacent inflammation/fluid, favored to represent appendicitis; a less likely differential diagnosis includes Meckel’s diverticulum. Lung fields on the CT scan were clear (Figures 1-2).

The pediatric surgery team was consulted and agreed the CT was consistent with appendicitis. The patient was admitted with IV antibiotics (ceftriaxone and metronidazole), and IV fentanyl for pain control. The next day the patient was taken for laparoscopic appendectomy and noted to have perforated appendicitis. Microscopic pathology report revealed mucosal necrosis and transmural infiltration of neutrophils with the formation of microabscess, extending to the serosa. Grossly and microscopic evidence of perforation was identified. Parasites are not seen. There was no evidence of fecolith, malignancy, or neoplasia. The gross and microscopic pathology report confirmed appendicitis. The patient received four additional days of IV antibiotics and was discharged home on four additional days of oral amoxicillin-clavulanate. On the first day home, the patient had some continued vomiting. However, vomiting resolved with smaller feeds, and the patient did not require re-admission or further ED visits.

## Discussion

Appendicitis is one of the most common surgical childhood emergencies and its prevalence peaks in the second decade of life. The incidence of pediatric appendicitis varies from 11 to 14 cases per 10,000, but fewer than 0.4% of cases occur in the first year of life [1,3-5]. The low incidence of appendicitis in this age group is thought to be due to the different structure of the appendix in the first year of life. Initially, the appendix is more funnel-shaped with less lymphoid tissue, leading to a lower risk for obstruction and development of appendicitis [3]. The appendix grows to the normal adult-like conical shape by one to two years of age with lymphoid follicle hyperplasia occurring until the late teenage years. Scientists believe that the infant's mainly soft solid diet also decreases the frequency of appendicitis in children < 1 year old. Although there are no congenital abnormalities that predispose infants to appendicitis, some case series report the rare occurrence of appendicitis in patients with an incarcerated inguinal hernia where part of the appendix is inside the hernia (Amyand's hernia) [3]. Appendicitis presentation without the presence of a hernia in early childhood is uncommon and usually atypical and delayed.

 Symptoms of appendicitis can be non-specific and can mimic other common pediatric diseases. In previous literature, early childhood appendicitis usually presents with vomiting (71-90%), pain (35-94%), diarrhea (18-57%), and fever (32-100%). Other nonspecific findings include irritability, refusal of feeds, and cough, or rhinitis [1,4,6-8]. Physical examination findings include a temperature > 38°C (87-100%), diffuse tenderness (55-92%), and abdominal distention (62-94%). Similar to patients in other cases reported, our patient also had fever, vomiting, irritability, and physical examination findings of abdominal pain. With these non-specific symptoms, more common causes of abdominal pain may be higher on the differential than appendicitis, such as gastroenteritis, otitis media, constipation, urinary tract infection, URI, or intussusception, causing further delay in diagnosis. URI or diarrheal symptoms at the time of presentation can anchor a differential diagnosis with a viral illness, deferring the final diagnosis [9-12]. One study showed that diagnosis for patients with symptoms of diarrhea was delayed by almost two days [11]. The patient in our case was diagnosed by a previous provider for otitis media and treated with intramuscular antibiotics, delaying the ultimate diagnosis of appendicitis.

Many other cases in the literature of infant appendicitis report a delay in diagnosis due to non-specific signs and symptoms at presentation. In children of three years or younger, studies show rates of missed diagnosis of 50-100% [1,2,13]. This finding compares to findings in older children (< 12 years old), for whom previous provider evaluation documents misdiagnosis in 25-58% of cases [1,2,11]. The normal non-verbal nature of young children, along with their inability to communicate the location of pain, significantly limits their ability to locate pain and infection. In children < 5 years old, the average time to diagnosis is around three to four days after the onset of symptoms, similar to our case [1,5,11,14]. One showed that the younger the patients were, the longer the delay in diagnosis, with the longest delay seen in the under-two-year-old group [4]. Appendicitis in infants is commonly diagnosed intraoperatively since the majority of their signs and symptoms are non-specific. 

Without a diagnosis of appendicitis, young children are at risk of developing worsening disease, intra-abdominal abscess, perforation, and bacteremia. Each day of delay in diagnosis doubles the risk of perforation in children < 5 years old [2,9]. Studies also show an increase in perforation for younger age, female sex, prior medical visits in the previous 48 hours, length of symptoms, fever, and presence of appendicolith [2,9]. Perforation rates in young children are 65-85% if < 5 years old, 84% < 3 years old, 93-100% < 2 years old, and 100% < 1 year old. These rates are all statistically higher than perforation rates in older children (> 5 years old) [4,6,7,9,15]. Some believe that the higher rate of perforation may be due to the lack of an adequate omental barrier from anatomic immaturity, while others believe it may be due to the difficulty in diagnosis in these patients [1]. Young children presenting with diarrhea or vomiting were noted to have higher rates of perforation, which may be due to anchoring of alternative diagnoses that caused a delay in the final diagnosis of appendicitis [4,15].

For young children who look ill, providers may order blood work to check for WBC and inflammatory markers, but this test can be non-specific. Studies show that only half of children with appendicitis will have an elevated WBC [8]. Previous case reports of children < 5 years old demonstrated neutrophilia with left shift seen in 80-100% of cases [7,8,11]. A large meta-analysis of pediatric and adult studies showed sensitivity highest for WBC (62%), followed by CRP (57%) and procalcitonin (33%). Specificity was shown to be highest for procalcitonin (89%), followed by CRP (87%) and WBC (75%) [16]. In young children (< 5 years old), procalcitonin had more specificity in appendicitis with perforation or complications [17]. Other studies showed that elevated WBC could not differentiate between appendicitis and perforated appendicitis [2,4]. New literature demonstrates that an elevated fibrinogen level can aid in the diagnosis of appendicitis in preschool-aged children presenting with abdominal pain, especially in complicated appendicitis [17,18]. These tests may not be available at all institutions and may require interpretation with clinical correlation.

Using examination findings and laboratory abnormalities in appendicitis clinical prediction scores in children < 4 years old were found to be inadequate to rule out appendicitis [19]. Overall, laboratory values do not have specificity for only appendicitis and may be elevated in other disease processes more common in this age range. One study analyzing the misdiagnosis of pediatric appendicitis (< 21 years old) recommended obtaining a WBC and ultrasonography for patients with at least intermediate risk of appendicitis [5].

Abdominal CT, with high sensitivity and specificity, is considered the radiographical gold standard to confirm the diagnosis of appendicitis. However, due to concerns about radiation exposure leading to a risk of increased lifetime cancer incidence, abdominal CT is not a first-line imaging modality in the workup for appendicitis. Many start with an abdominal radiograph (X-ray), which can be useful to exclude alternative diagnoses such as lower lobe pneumonia or severe constipation but rarely provides a diagnosis since fecalith is seen only in < 9% of patients [4]. US is an alternative that provides an easily available, low-cost diagnostic test without ionizing radiation, with the caveat that it is operator-dependent. Some studies of children < 3 years old with atypical symptoms of appendicitis showed sensitivity to US (95-98%), while others have shown US to be inconclusive in most of their patients [2,5,12,19,20]. This discrepancy indicates the operative dependency of US. Because abdominal US can miss cases of appendicitis, it is reasonable to consider more advanced imaging such as an abdominal CT scan when the US is inconclusive to gain increased diagnostic sensitivity [20]. In our case study, the patient did obtain a US that did not visualize the appendix or show any abnormal echogenic mesenteric fat or mass. Due to concern for guarding on abdominal examination, the patient obtained an abdominal CT with oral and IV contrast. The results were concerning for appendicitis that was confirmed intraoperatively. 

All children with appendicitis require admission for IV antibiotics with surgical consultation. Complications in children, such as perforation, are associated with longer lengths of stay [4,9,15]. Very young children demonstrate higher rates of delay in diagnosis and higher rates of perforation, leading to prolonged hospital stays (5-15 days) [1,4,11,12]. Due to the high rate of perforation in infants at presentation and the paucity of diagnosis pre-operatively, the consensus in the literature recommends prompt surgical interventions for infants with appendicitis. Emerging literature describes lower complication rates with conservative appendectomy, with the tradeoff of longer hospital stays and more days on IV antibiotics [5]. Surgical subspecialists must weigh the risks and benefits of emergent surgical interventions with the need for prolonged hospitalization and IV antibiotics. The patient in our case was admitted for five days total for IV antibiotics.

Despite alternative reasons for clinical presentation (i.e., otitis media, vomiting, viral illness), diagnosis, in this case, was achieved through serial abdominal examinations and a broad differential diagnosis resulting in further imaging. Emergency physicians evaluating a patient with a previous diagnosis must navigate the pertinent history, physical examination findings, and overall appearance of the patient to prevent anchoring on the first diagnosis.

## Conclusions

This case illustrates how easy it is to misdiagnose infant appendicitis due to factors including normal developmental speech barriers, nonspecific presentations, and overlap of symptoms with many other common childhood illnesses, along with a challenging abdominal examination. Delay in diagnosis leads to increased rates of perforation and complications. It is important for providers to trust abnormal physical examination findings, especially abdominal guarding against palpation, and maintain a wide differential diagnosis, without anchoring on previous diagnoses, in order to identify appendicitis in young children. Overall, though appendicitis may be uncommon in this age group, it is not so rare that we can overlook it in a differential diagnosis.
